# Gypenoside IX restores Akt/GSK-3β pathway and alleviates Alzheimer’s disease-like neuropathology and cognitive deficits

**DOI:** 10.18632/aging.205295

**Published:** 2023-12-12

**Authors:** Ling Lei, Yong Luo, Dongkun Kang, Fumin Yang, Dongli Meng, Jian-Zhi Wang, Rong Liu, Xiaochuan Wang, Hong-Lian Li

**Affiliations:** 1 School of Basic Medicine, Key Laboratory of Education Ministry/Hubei Province of China for Neurological Disorders, Tongji Medical College, Huazhong University of Science and Technology, Wuhan 430030, China; 2 Co-Innovation Center of Neuroregeneration, Nantong University, Nantong, Jiangsu 226001, China; 3 Shenzhen Huazhong University of Science and Technology Research Institute, Shenzhen 518000, China

**Keywords:** Gypenoside IX, anti-apoptotic, Aβ, tau, Akt/GSK-3β

## Abstract

The main pathological changes of Alzheimer's disease (AD), a progressive neurodegenerative disorder, include senile plaque (deposited by amyloid beta), neurofibrillary tangle (formed by paired helical filaments composed of hyperphosphorylated tau), and massive loss of neurons. Currently there is a lack of ideal drugs to halt AD progression. Gypenosides (GPs), a kind of natural product, possesses potential therapeutic effects for neurodegenerative diseases, including AD. However, the specific role and mechanism of GPs for AD remain unclear. In the current study, we used staurosporine (STP), an inducer of apoptosis and causing tau hyperphosphorylation, to mimic AD models, and explored the role and mechanism of Gypenoside IX (one of the extracts of Gynostemma, GP for short name in our experiments) in STP treated primary hippocampal neurons and rats. We found STP not only increased apoptosis and tau hyperphosphorylation, but also significantly increased Aβ production, resulting in synaptic dysfunction and cognitive decline in mimic AD models by STP. GP was found to rescue apoptosis and cognitive impairments caused by STP treatment. Moreover, GP recovered the decreased synaptic proteins PSD95, Synaptophysin and GluR2, and blocked dendritic spine loss. Interestingly, GP decreased the STP induced tau hyperphosphorylation at different sites including S-199, S-202, T-205, T-231, S-262, S-396, and S-404, and at the same time decreased Aβ production through down-regulation of BACE1 and PS1. These effects in STP treated primary hippocampal neurons and rats were accompanied with a restoration of AKT/GSK-3β signaling axis with GP treatment, supporting that dysregulation of AKT/GSK-3β pathway might be involved in STP related AD pathogenesis. The results from our research proved that GP might be a potential candidate compound to reduce neuronal damage and prevent the cognitive decline in AD.

## INTRODUCTION

Alzheimer’s disease (AD), a progressive neurodegenerative disease, is clinically characterized by decline in cognitive function [1]. Nowadays, the AD mechanism is still unknown. Extracellular senile plaques (SPs), intracellular neurofibrillary tangles (NFTs), and massive loss of neurons are primary hallmarks in AD patients’ brains [2–4]. SPs are formed by deposits of Amyloid beta (Aβ) peptide [5, 6]. Hyperphosphorylated tau aggregates and forms NFTs in the involved neurons [7]. Abnormal hyperphosphorylated tau is due to the overactivation of protein kinases or/and the inactivation of phosphates. SPs and NFTs all lead to structural damage of neurons and synapses, resulting in neuronal loose, ultimately decreased memory and learning of AD [7–9].

AD is mainly sporadic, and the pathogenesis is very complex [10]. Although scientists have made a great number of efforts, there has yet to be an ideal drug to cure or simply stop the development of AD. Gypenosides (GPs) have been used in Chinese folk medicine for many years. Accumulating data have shown that GPs possess neuroprotective effects, including anti-inflammation [11], antioxidative stress [12], and antineuronal apoptosis effects [13]. Moreover, GPs treatment has been a therapeutic strategy in stroke and traumatic brain injury in China [14–16]. We speculated that maybe GPs alleviate the symptoms of AD and slow down the progression. To explore this hypothesis, we intended to use Gypenoside IX (GP for short name in our experiments), one of the main extracts from Gynostemma to treat AD cell and animal models.

Staurosporine (STP) is commonly used as an inducer of apoptosis [17, 18]. Our previous study has shown that stereotactic injections in the hippocampus of rats with STP induced neuronal apoptosis and caused an increased tau phosphorylation [19], finally leading to cognitive impairments [20–22]. Therefore, in the present study, primary hippocampal neurons treated with STP were used as AD cell models, and rats injected with STP in bilateral hippocampi as AD animal models. Then, we investigated the effects of GP on STP treated cell and rat models. We found that GP rescues STP induced AD pathologies and cognitive impairments. Moreover, GP treatment recovered AKT/GSK-3β signaling pathway in STP cell and rats. Hence, our results reveal GP as a promising drug for reducing the symptoms of AD.

## RESULTS

### GP attenuated cytotoxicity and apoptosis in primary hippocampal neurons treated with STP

**Figure 1:**
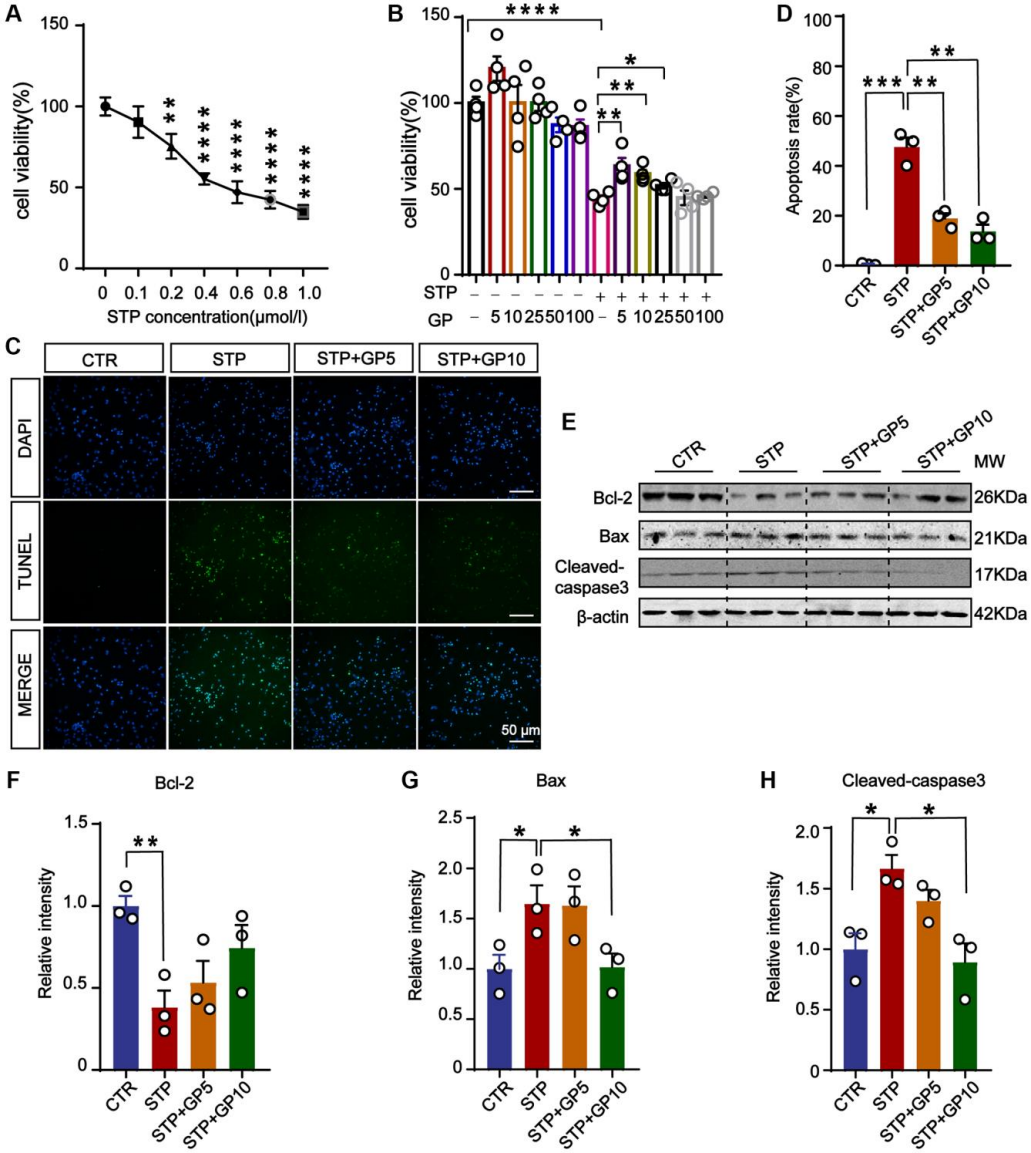
**GP attenuated STP-induced cytotoxicity and inhibited apoptosis in primary hippocampal neurons.** (**A**) STP inhibited cell viability in a dose-dependent manner. 0.4 μM of STP caused a decline of 50% in cell viability. *n* = 4. (**B**) GP rescued the cell viability with different concentrations (5–25 μM). *n* = 4. (**C**) TUNEL assay verified the inhibition of STP-induced (0.4 μM) apoptosis by GP (5 μM and 10 μM). Scale bar = 50 μm. (**D**) Quantification of the TUNEL/DAPI using ImageJ software. (**E**–**H**) Western blots and quantification of the relative protein expression levels (Bcl-2, Bax, and cleaved-caspase-3) after normalization to the β-actin signal. Data represent mean ± SEM, *p*-value significance is calculated from one-way ANOVA, *n* = 3. ^*^P < 0.05, ^**^P < 0.01, ^***^P < 0.001, ^****^P < 0.0001.

To detect the toxic effects of STP on cells and select the appropriate concentration for cytotoxicity model, we treated primary hippocampal neurons with different concentrations of STP (Figure 1A). It showed that STP led to a decrease in cell viability. When the STP concentration was 0.4 μM, the cell viability approximately decreased by 50%. On the basis of previous report [23], we chose 0.4 μM STP as ideal concentration to make cell apoptosis in the following experiments. To test the effect of GP on neuronal toxicity, the primary hippocampal neurons were incubated with GP with or without STP for 24 h (Figure 1B). The CCK8 assay showed that 5 μM and 10 μM GP rescued cell viability decreased by STP, while GP had no obvious cytotoxic effect on hippocampal neurons. The result from TUNEL assay also supported that GP significantly attenuated STP-induced cell death (Figure 1C, 1D). We further checked the alterations of apoptotic proteins, and found that STP activated proapoptotic Bax and cleaved-caspase-3 [24, 25]. Meanwhile anti-apoptotic protein Bcl-2 was markedly decreased. Interestingly, GP10 recovered the expression levels of those above apoptotic proteins (Figure 1E–1H). Taking together, these findings showed that GP has neuroprotective effect on the neurons with reduced survival caused by STP, and reduces their apoptosis by restoring the expression of cleaved-caspase-3, Bax and Bcl-2.

### GP attenuated STP-induced tau phosphorylation and reduced Aβ production in primary hippocampus neurons

**Figure 2:**
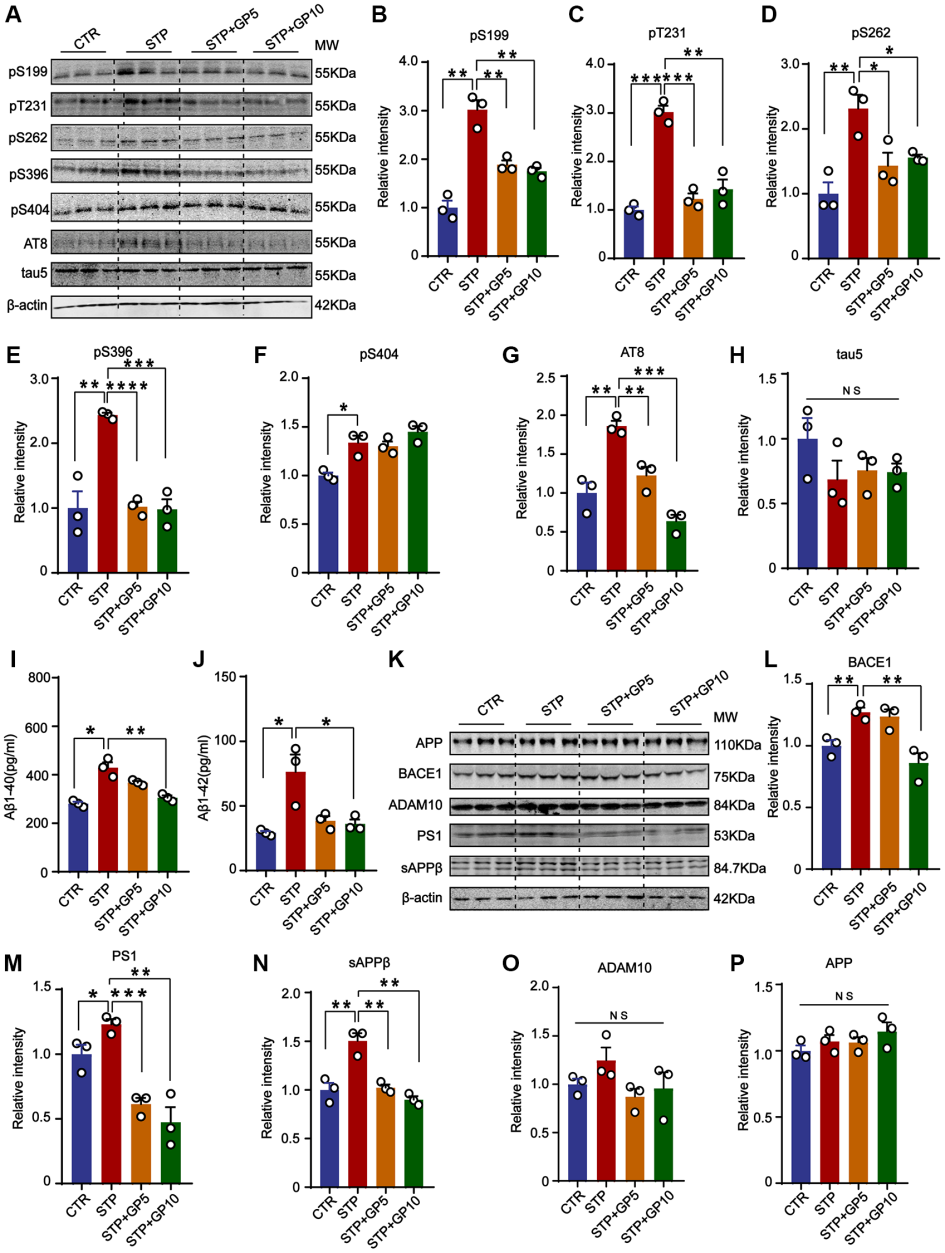
**GP attenuated STP-induced tau phosphorylation and reduced Aβ generation in primary hippocampus neurons.** (**A**) Western blot for tau phosphorylation levels at the sites of pS199, pT231, pS262, pS396, pS404, AT8 and total tau (Tau-5) in primary hippocampus neurons. (**B**–**H**) Quantification of the relative protein expression levels (pS199, pT231, pS262, pS396, pS404, AT8 and Tau-5) after normalization to the β-actin signal. (**I**, **J**) ELISA assay of the levels of Aβ40 and Aβ42 in rat primary hippocampal neurons. (**K**) Western blots for APP, BACE1, ADAM10, PS1 and sAPPβ in rat primary hippocampal neurons. (**L**–**P**) Quantification of the relative protein levels (BACE1, PS1, sAPPβ, ADAM10 and APP) after normalization to the β-actin signal. Data represent mean ± SEM, *p*-value significance is calculated from one-way ANOVA, *n* = 3. ^*^P < 0.05, ^**^P < 0.01, ^***^P < 0.001 and ^****^P < 0.0001. Abbreviation: NS: no significance.

To investigate the effect of GP on tau phosphorylation, Western blot was carried out (Figure 2A). We found that tau phosphorylation levels at pS199, pS202/ pT205(AT8), pT231, pS262, pS396, and pS404 were significantly increased in STP-treated primary hippocampal neurons, and GP treatment effectively blocked STP-induced tau hyperphosphorylation at all above sites except pS404 (Figure 2B–2H). Together with previous studies showing GSK-3β association with tau phosphorylation at these sites [26, 27], we speculate that GP treatment probably alleviates STP-related tau phosphorylation by activation of GSK-3β.

To further study the effect of GP on Aβ production, we detected the levels of Aβ40 and Aβ42. The ELISA showed that both Aβ40 and Aβ42 were obviously increased in STP group, and GP treatment restored the changes (Figure 2I, 2J). Aβ peptides are generated from APP cleavage by β-secretase (BACE1) and γ-secretase [28]. As the catalytic subunit of γ-secretase, Presenilin-1 (PS1) mediates cleavage of type I transmembrane proteins APP [29]. ADAM10 (alpha-secretase of APP) is mainly responsible for a non-toxic Aβ peptide generation [30]. Next, we measured the levels of BACE1, AMAD10, PS1, and sAPPβ (Figure 2K), and found that the levels of BACE1, PS1 and sAPPβ were significantly increased by STP, but not in GP treated primary hippocampus neurons. Only 5 μM GP did not inhibit the increasing of BACE1 level by STP (Figure 2L–2N). Meanwhile, the levels of APP and ADAM10 among these groups had no change (Figure 2O, 2P). The data showed that GP attenuated Aβ generation through partially regulating BACE1 and γ-secretase expression level in STP treated cell models.

### GP restored the activity of AKT/GSK-3β signaling axis

**Figure 3:**
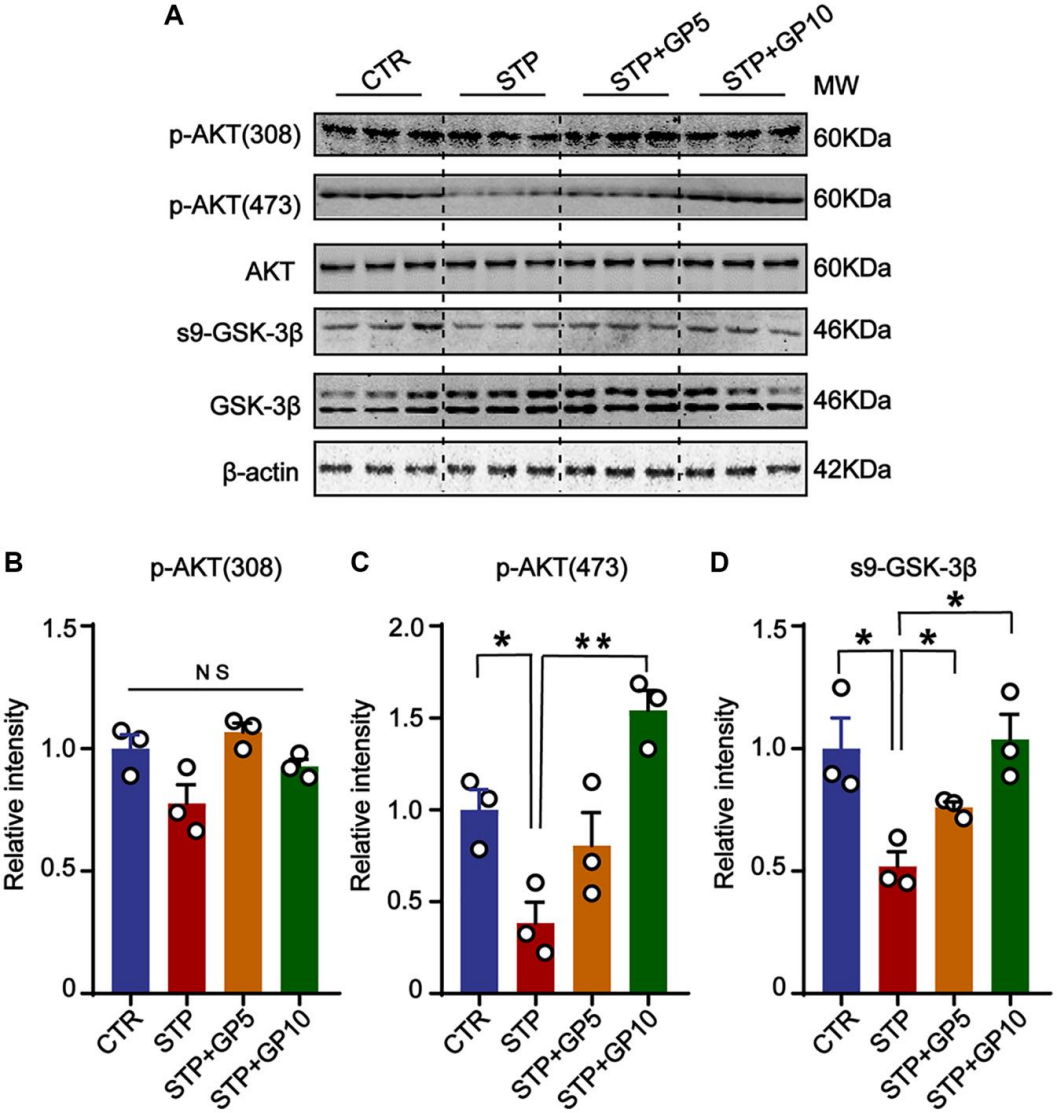
**GP restored the activity of AKT/GSK-3β signaling axis.** (**A**) Western blots for AKT/GSK-3β pathway-related proteins. (**B**–**D**) Quantification of the relative protein levels of p-AKT (308), p-AKT (473) and s9-GSK-3β after normalization to the β-actin signal. Data represent mean ± SEM, *p*-value significance is calculated from one-way ANOVA, *n* = 3. ^*^P < 0.05, ^**^P < 0.01. Abbreviation: NS: no significance.

Many previous researches revealed that AKT/GSK-3β signaling pathway had important functions related with apoptosis, neurogenesis, mitophagy, and synaptic plasticity in the central nervous system [31, 32]. Accumulating data reported that many natural products protect synaptic function, and inactive microglia through the AKT/GSK-3β axis in AD model [33, 34]. We here suppose that GP-mediated neuroprotection might be associated with AKT/GSK-3β pathway. In hippocampal neurons, we detected the active forms of AKT (phosphorylated AKT at Thr308 and Ser473) [35], and found that 5 μM and 10 μM, both concentration of GP rescued the STP-induced decline of p-AKT (473), but not p-AKT (308) (Figure 3A–3C). GSK-3β, a major kinase of tau [36], and also a regulator of Aβ generation [37], is inactive by phosphorylated at Ser9 [38]. Consistent with the change of p-AKT, STP promoted GSK-3β activity by decreasing the level of Ser9 phosphorylation, which was blocked by GP (Figure 3A, 3D). This result suggested that GP restored the Akt/GSK-3β axis and might be associated with an anti-apoptotic effect.

### Effects of GP on neuronal dendrites and synaptic proteins in primary hippocampal neurons

**Figure 4:**
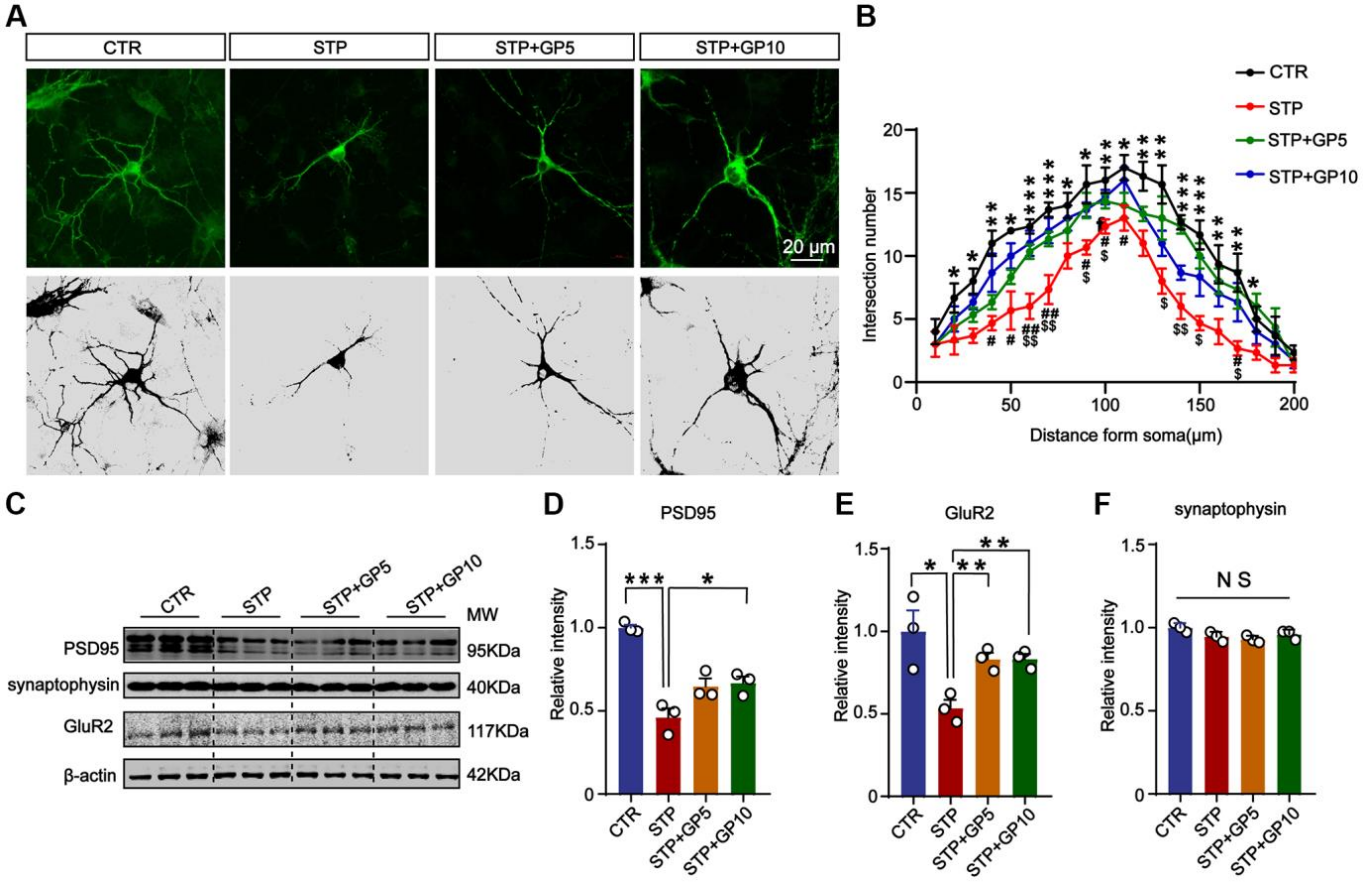
**Effects of GP on neuronal dendrites and synaptic plasticity-related proteins in the hippocampus neurons.** (**A**) Representative images of immunofluorescence staining for MAP-2 labeled neuronal dendrites; scale bar = 20 μm. (**B**) Sholl analysis of dendritic arborization of neurons in (**A**). (**C**) Representative Western blots of PSD-95, synaptophysin, and GluR2. (**D**–**F**) Quantitative analysis of PSD-95, GluR2, and synaptophysin after normalization to the β-actin signal. Data represent mean ± SEM, *p*-value significance is calculated from one-way ANOVA, *n* = 3. ^*^P < 0.05, ^***^P < 0.001 vs. control group, NS, no significance. ^#^*P* < 0.05, ^##^*P* < 0.01 vs. GP10, ^$^*P* < 0.05, ^$$^*P* < 0.01 vs. GP5.

The destruction of neuronal structure will affect the structure and function of synapses, and ultimately destroy neural networks. Next, to investigate whether GP could ameliorate synaptic damage induced by STP, we examined the morphology of cultured hippocampal neurons and the levels of synaptic proteins. Immunofluorescence staining and Sholl analysis showed that MAP-2 labeled neuronal dendrites and branches significantly decreased in STP groups, while GP treatment obviously alleviated the damage of neuronal dendrites in STP treated neurons (Figure 4A, 4B). Next, we detected the synaptophysin, PSD-95, GluR2 (Figure 4C). The levels of PSD-95 and GluR2 in STP group were markedly decreased when compared with control. But GP obviously elevated the levels of these proteins in STP+GP groups (Figure 4D, 4E), while the levels of synaptophysin proteins was unchanged (Figure 4F). This finding suggested that GP protected STP-induced synaptic dysfunction.

### GP improves learning and memory impairment in STP-treated rats

**Figure 5:**
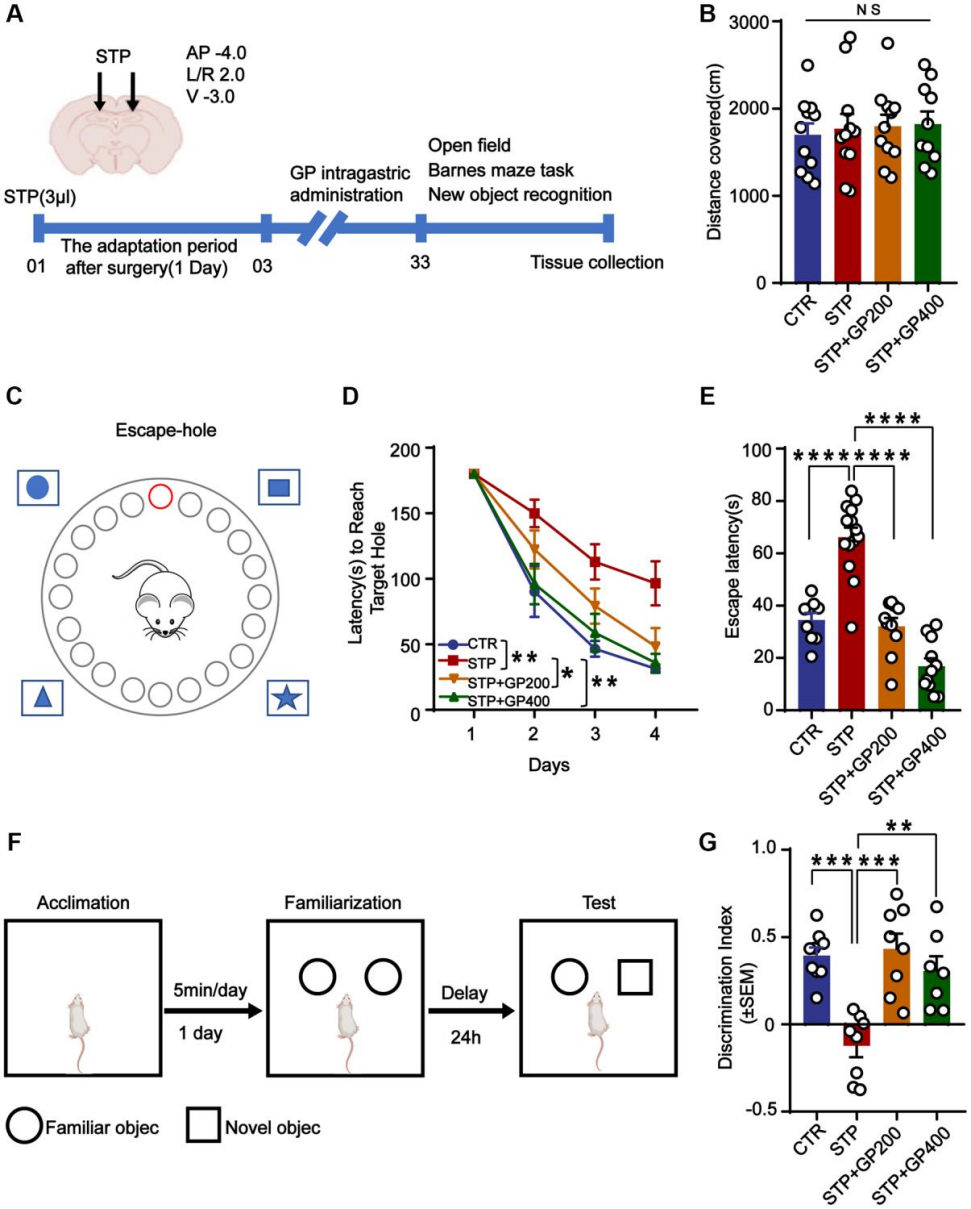
**GP improves learning and memory impairment in STP-treated rats.** (**A**) Study design timeline performed in the study. (**B**) The total distance was analyzed in the open-field test. (**C**–**D**) Escape latency in 4 days was analyzed in the Barnes maze test. (**E**) Escape latency on test day 5 was analyzed in the Barnes maze test. (**F**, **G**) The novel object recognition (NOR) test showed the measured recognition index of the new object within 24 hours. Data represent mean ± SEM, *p*-value significance is calculated from one-way ANOVA. ^*^p < 0.05, ^**^p < 0.01, ^***^P < 0.001 and^ ****^P < 0.0001, Abbreviation: NS: no significance. *n* represents the number of rats in each group. For (**B**), Control *n* = 11, STP *n* = 11, STP+GP200 *n* = 11, STP+GP400 *n* = 10. For (**D**, **E**), Control *n* = 9, STP *n* = 14, STP+GP200 *n* = 10, STP+GP400 *n* = 11. For (**B**), Control *n* = 9, STP *n* = 8, STP+GP200 *n* = 8, STP+GP400 *n* = 7.

It was demonstrated that GP could resist or reduce AD pathologies and synaptic dysfunction caused by STP in primary neurons. However, whether STP induces these harmful effects, or even leads to cognitive defects *in vivo*, and whether GP blocks them are unclear. To address these points, we designed the animal experiments, dividing the rats in four groups according to the schematic diagram in Figure 5A (see details in material and methods part).

The open-field test showed no significance difference in total distance among these groups (Figure 5B), implying that neither STP nor GP affected locomotion activity. In the Barnes maze (the principle and method being shown in Figure 5C) experiment, as the training days increased, the escape latency of rats in all groups decreased, indicating that all the rats got certain memories as practicing for more days. However, the escape latency of rats in the STP group was markedly higher compared to that of control. Whereas the escape latency of rats treated with STP+GP was significantly reduced. (Figure 5D). Furthermore, STP group showed obviously increased exploration times before finding the hidden box, which was significantly decreased in the GP administration group on test day 5 (Figure 5E). Next, we carried out NOR and found that the curiosity toward exploring new things of STP rats was markedly reduced compared to control. However, GP blocked the decreased time spent for exploration of new object by STP (Figure 5F, 5G). Together, the data suggested that GP ameliorated cognitive impairment in STP rats.

### Effect of GP on neuronal apoptosis in STP rats

**Figure 6:**
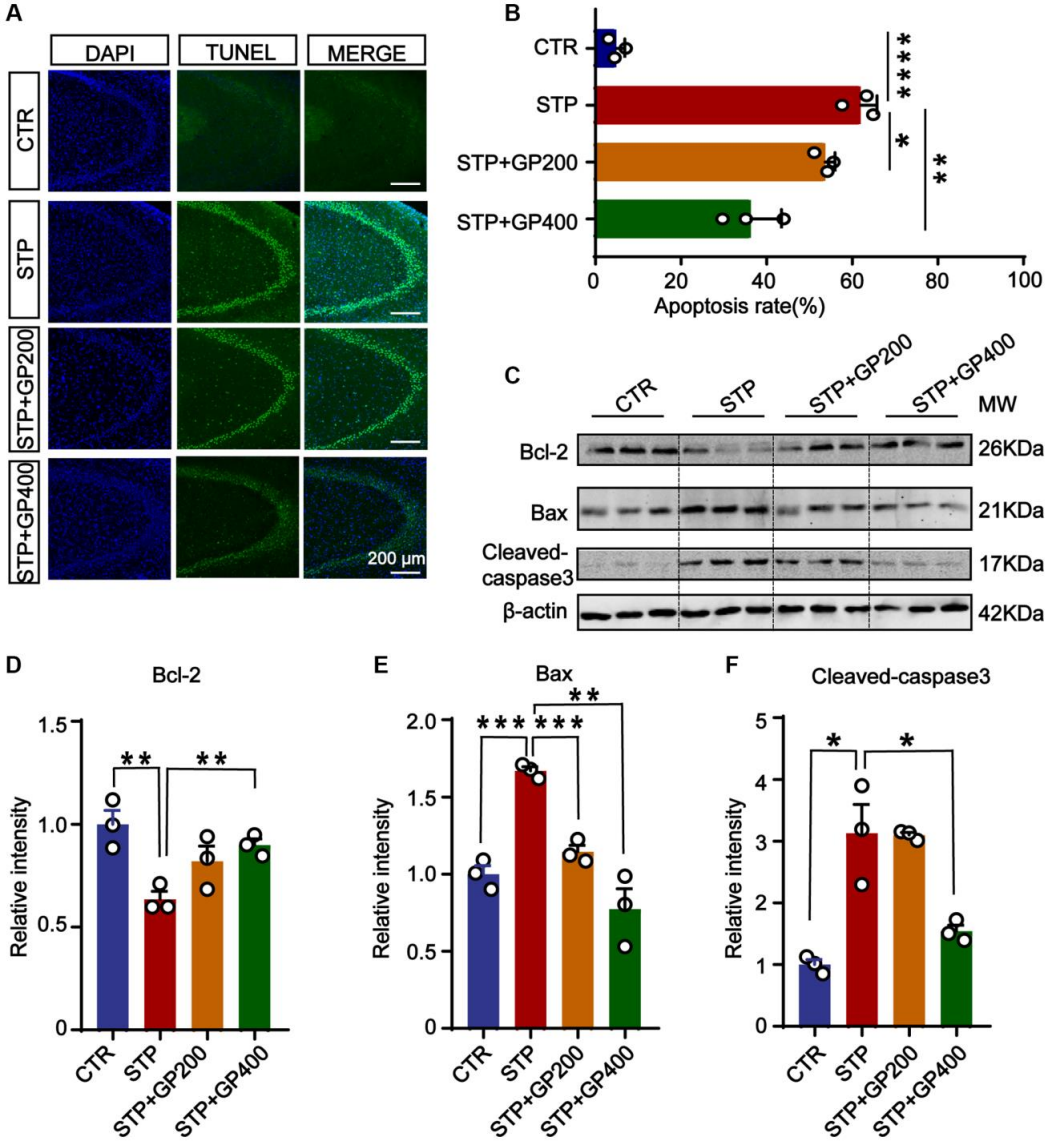
**Effect of GP on neuronal apoptosis in the hippocampus of STP Rats.** (**A**) Representative TUNEL-positive cells (green) in hippocampal CA3 region. Nuclei were stained with DAPI (in blue). Scale bar = 200 μm. (**B**) Quantification of the TUNEL/DAPI using ImageJ software. (**C**–**F**) Representative images and quantitative analysis of Western blotting for Bcl-2, Bax, and caspase-3 in the hippocampus after normalization to the β-actin signal. Data represent mean ± SEM, *p*-value significance is calculated from one-way ANOVA, *n* = 3. ^*^p < 0.05, ^**^p < 0.01, ^***^P < 0.001 and ^****^P < 0.0001.

To investigate the effect of GP on apoptosis *in vivo*, we performed the TUNEL staining to test the apoptotic rate in the hippocampi of four groups. As shown in Figure 6A, 6B, in the brain slices of the STP group, the neuronal apoptotic rate was higher when compared to control, especially in the CA3 of hippocampus. Interestingly, both GP200 and GP400 significantly reduced the apoptosis. Moreover, STP treatment upregulated Bax and cleaved caspase-3, and downregulated Bcl-2, which were blocked by GP (Figure 6C–6F). These data further supported that GP might prevent from apoptosis and protect the brain neurons in STP-induced AD rats.

### GP blocks tau hyperphosphorylation and Aβ overproduction in STP rats

**Figure 7:**
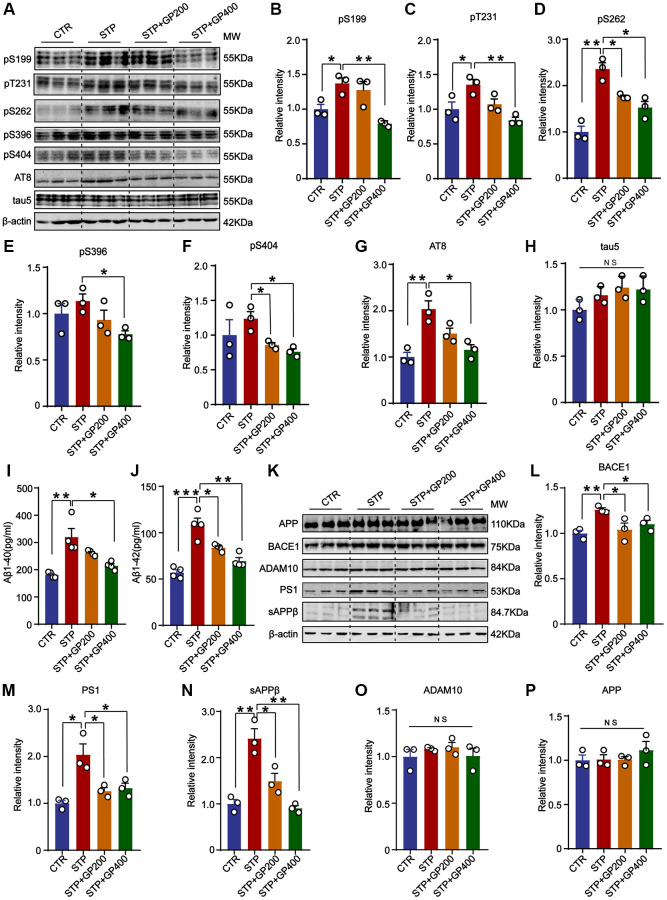
**GP blocks tau hyperphosphorylation and Aβ overproduction in STP rats.** (**A**) Western blot for tau phosphorylation levels at the sites of pS199, pT231, pS262, pS396, pS404, AT8 and total tau (tau-5) in rat hippocampus. (**B**–**H**) Quantification of the relative protein expression levels (pS199, pT231, pS262, pS396, pS404, AT8 and tau-5) after normalization to the β-actin signal. (**I**, **J**) ELISA assay of the levels of Aβ40 and Aβ42 in the rat hippocampus. (**K**) Western blots for APP, BACE1, ADAM10, PS1 and sAPPβ in rat hippocampus. (**L**–**P**) Quantification of the relative protein levels (BACE1, PS1, sAPPβ, ADAM10 and APP) after normalization to the β-actin signal. Data represent mean ± SEM, *p*-value significance is calculated from one-way ANOVA, *n* = 3. ^*^p < 0.05, ^**^p < 0.01 and ^***^P < 0.001, Abbreviation: NS: no significance.

Tau hyperphosphorylation and Aβ toxicity contribute to cognitive impairment in AD. In the primary hippocampal neurons experiment, we found that GP reduced tau phosphorylation levels and inhibited STP-induced Aβ overproduction (Figure 2). Thereby, we further detected tau phosphorylation *in vivo.* The results indicated that compared to that of control rats, tau phosphorylation level in the hippocampus of the STP rats was increased at Ser199 (pS199), Thr231 (pT231), Ser262 (pS262), and AT8 (Ser202, Thr205), but not at Ser396 (pS396) and Ser404 (pS404) site (with only an increasing trend). Supplementation with GP in STP rats effectively restored tau phosphorylation (Figure 7A–7H).

Next, we studied the effect of GP on APP metabolism in STP rat. Firstly, we carried out the ELISA and found that GP200 and GP400 decreased the Aβ40 and Aβ42 overproduction induced by STP (Figure 7I, 7J). Then, we investigated the possible reason why GP reduces Aβ production, we detected the impact of GP on APP processing and found that there was no significant increase in APP level in the hippocampus of STP rats compared to control (Figure 7K, 7P). The APP cleavage enzymes and cleavage fragments were detected in rat brain (Figure 7K). Compared to control, the protein levels of BACE1, PS1, and sAPPβ in the hippocampus of STP rats were significantly higher, which was blocked by GP treatment. However, ADAM10 was not differentially expressed in the hippocampus of the four groups of rats (Figure 7L–7O). These data further supported that GP significantly inhibited STP-induced tau hyperphosphorylation and Aβ overproduction.

### GP reactivates AKT/GSK-3β pathway in STP rats

**Figure 8:**
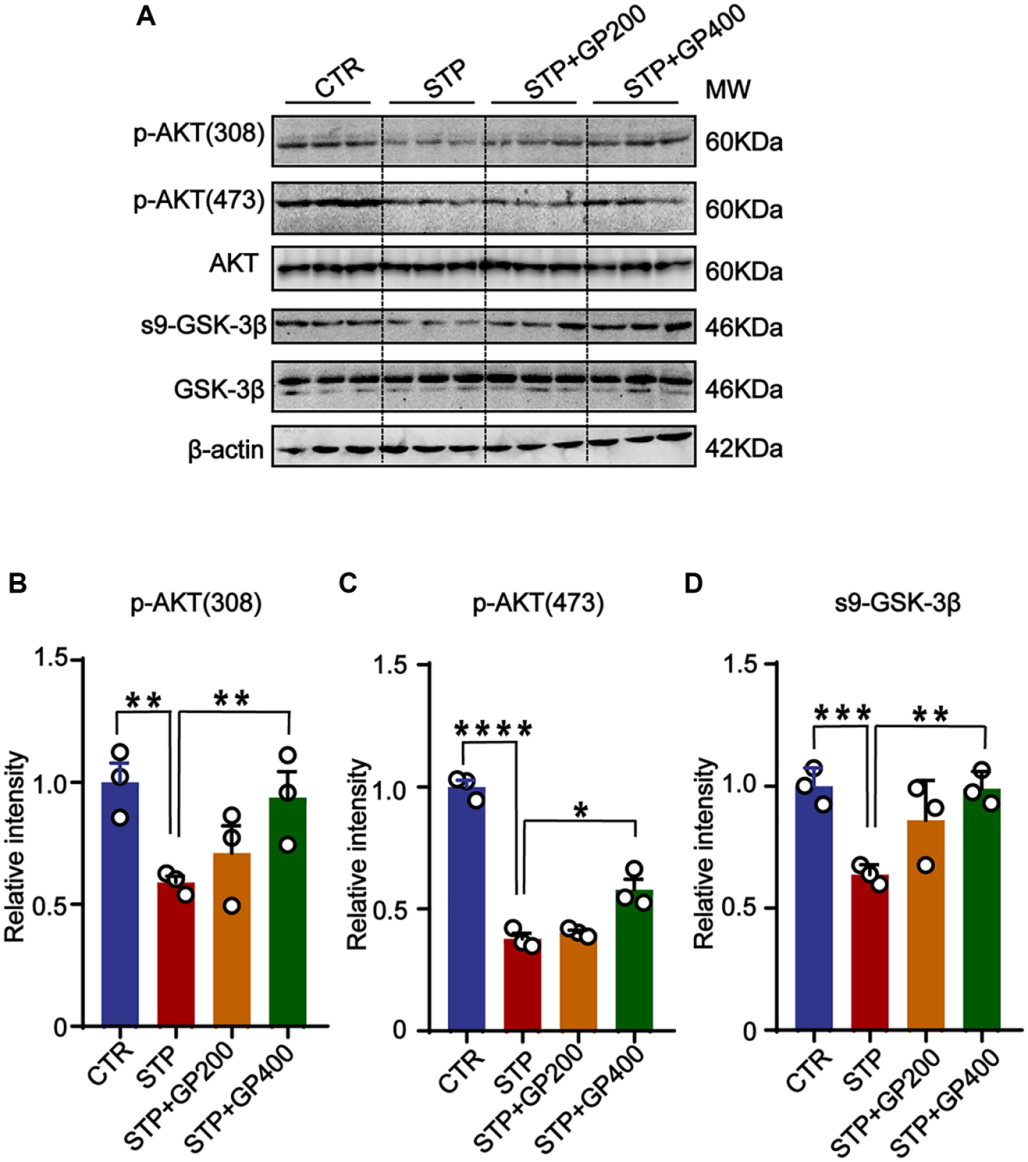
**GP reactivates AKT/GSK-3β pathway in STP rats.** (**A**) Western blots for AKT/GSK-3β pathway-related proteins. (**B**–**D**) Quantification of the relative protein levels (p-AKT (308), p-AKT (473), s9-GSK-3β) after normalization to the β-actin signal. Data represent mean ± SEM, *p*-value significance is calculated from one-way ANOVA, *n* = 3. ^*^P < 0.05, ^**^P < 0.01, ^***^P < 0.001 and ^****^P < 0.0001.

To further examine if GP affects AKT/GSK-3β activity *in vivo*, we detected p-AKT (308), p-AKT (473) and s9-GSK-3β expression in four groups. The levels of p-AKT (T308/S473) and s9-GSK-3β in STP rats were markedly decreased compared to that of control, while the expressions of p-AKT (T308/S473) and s9-GSK-3β were increased after treatment with GP (Figure 8A–8D). These finding suggested that the AKT/GSK-3β axis might be associated with the STP-related AD pathologies and cognitive impairments.

### GP recovers synaptic deficit in STP rats

**Figure 9:**
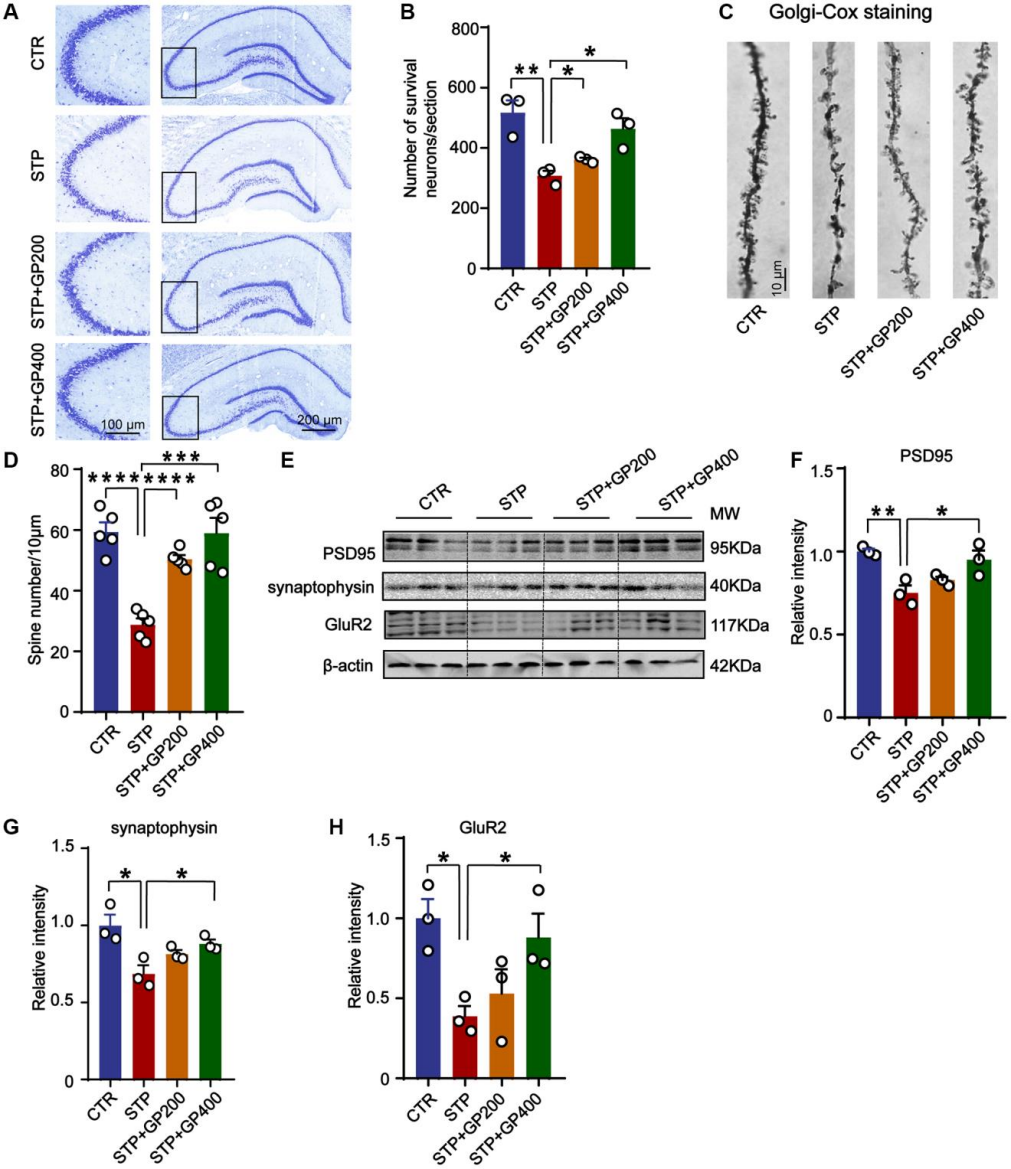
**GP reverses dendritic spine loss and recovers postsynaptic proteins.** (**A**, **B**) Representative Nissl staining images and the quantification of neuron number in the CA3 region of rat hippocampus, scale bar = 200 and 100 μm for low and high magnifications respectively. (**C**, **D**) Representative Golgi staining images and statistics analysis of spine number in the dentate gyrus region of rat hippocampus (scale bar, 10 μm). *n* = 5. (**E**–**H**) Western blots and quantitatively analysis for synaptic proteins (PSD95, GluR2 and synaptophysin) levels in rat hippocampus. Data represent mean ± SEM, *p*-value significance is calculated from one-way ANOVA, *n* = 3, ^*^p < 0.05, ^**^p < 0.01, ^***^P < 0.001 and ^****^P < 0.0001.

Since synaptic deficit is a pathological phenotype in AD [39], we evaluated the neuron and dendritic spine (where synapses being formed) lose in four groups of rats. Firstly, in CA3 of hippocampus in STP group, neuronal loss increased revealed by Nissl staining (Figure 9A, 9B). Meanwhile, STP treatment led to dendritic spine loss in the same region, and GP200 or GP400 obviously attenuated the spine loss (Figure 9C, 9D). Moreover, the levels of synaptic proteins including PSD95, synaptophysin and GluR2 were reduced in STP rats after treatment with GP200 or GP400 (Figure 9E–9H). These findings indicated that GP rescued synaptic damage in STP rats through recovering dendritic morphology and postsynaptic related protein.

## DISCUSSION

Alzheimer’s disease is a most common neurodegenerative disease [1, 6, 40]. It is classified as familial or sporadic. Dominant familial or autosomal dominant inheritance accounts for 1–5% of the clinic cases. It is classified as early-onset (EOAD; age <65 years) and manifests as mutations in the *PSEN1*, *PSEN2* or *APP* genes [41]. The characteristic pathological changes in Alzheimer’s disease include neuronal loss, extracellular deposition of Aβ plaques, and neuronal fiber tangles formed by intracellular tau protein hyperphosphorylation [42, 43]. The pathogenesis of AD is very complex, which leads to the slow development of drugs for its treatment. Several drugs targeting Aβ and phosphorylation of tau were discontinued due to poor efficacy [44, 45]. Using herbal medicine acts as an important therapeutic strategy in China. It may be hopeful to find useful agents from natural herbs to treat or stop the progression of AD. Gynostemma mainly distributed in China in Hunan, Hubei, Yunnan, Guangxi and other provinces, known as the “southern ginseng”, growing in the south of the Gynostemma medicinal content is relatively high, the folk said it is a magical “longevity herb”, which has been used widely for nervous system diseases [46]. The effect of Gypenosides extracted from Gynostemma in AD was explored by several researches. Some studies have reported that Gypenoside attenuates Aβ toxicity such as Aβ-induced inflammation, parallel autophagic and apoptotic cell death [47, 48]. By network pharmacology analysis, one work revealed that three compounds (GPM2, 7 and 9) in 13 active compounds of Gynostemma constructed network with eighteen intersecting targets of AD, in which HIF-1 signaling pathway and cytokine-cytokine receptor interaction were involved in the therapeutic effect [49]. These studies have identified the promising value of Gynostemma in the treatment of AD, but have not made it clear whether it can alleviate the major pathological changes and cognitive impairments in AD. Hence, in the current study we try to evaluate the effect of GP (Gypenoside IX, one of the main extracts from Gynostemma) on AD models.

Previous studies have found that STP can cause neuronal apoptosis, tau hyperphosphorylation, most important pathological characteristics in AD [19, 50, 51]. Therefore, in our experiments, we treated primary hippocampal neurons with STP and injected STP into rat hippocampus respectively, to mimic multiple AD pathological features simultaneously.

In the cell experiment, we found that STP significantly increased the apoptosis rate of primary hippocampal neurons through TUNEL method, and GP decreased the apoptosis rate. By Western blotting, it showed that GP reduced the increased Bax and cleaved caspase-3 caused by STP, but increased the decreased Bcl-2, further supporting that GP blocks STP-induced apoptosis. At the same time, STP treatment resulted in hyperphosphorylation of multiple sites of tau protein, increasing intracellular Aβ production and enzymes associated with APP cleavage, while GP treatment could obviously reverse these pathological changes. Consistent with previous studies reported that both tau hyperphosphorylation and apoptosis are associated with synaptic damage [52, 53], we also found that STP-induced tau hyperphosphorylation and apoptosis were accompanied with dendritic spine loss and synaptic proteins deficiency. Moreover, all STP-induced harmful effects were significantly mitigated by GP treatment in cultured primary neurons and rats. Behavioral tests also verified that GP improved the learning and memory in STP-caused AD rats. However, we found that GP+200 and GP+400 *in vivo* experiments could not completely reverse the neuronal apoptosis caused by STP, while the GP+400 animals took markedly shorter time to find the platform than the control group. We speculate two things might be relevant. The first is the complexity of drug interactions on higher concentration which should be clarified in the future study. The second possibility is that the loss of nerve cells due to apoptosis is not enough to cause cognitive dysfunction. Akt/GSK-3β pathway is closely to take part in many physiological processes. Dysregulation of this pathway has been implicated in various diseases, including cancer, neurodegenerative disorders, and metabolic disorders [54]. Akt phosphorylates GSK-3β at Serine 9 and in turn inactivates GSK-3β [54, 55]. Activation of GSK-3β is engaged in the regulation of amyloidogenic processing of APP, which generates Aβ [37, 56, 57]. As a therapeutic target, blocking GSK-3β attenuates the generation of Aβ peptides and its toxicity in neurons [58, 59]. As a tau kinase, the increase of GSK-3β in STP mice and rat promotes tau phosphorylation [19], and the blockage of GSK-3β attenuates tau phosphorylation [60, 61]. We here investigate the Akt/GSK-3β signaling axis, and found that both pS473-Akt and pS9-GSK-3β were significantly decreased in the brain of STP rats. GP treatment restored the levels of pS473-Akt and pS9-GSK-3β in STP-treated neurons and rats, supporting that GP attenuates STP-induced tau hyperphosphorylation and Aβ production might be associated with restoration of Akt/GSK-3β axis.

## CONCLUSION

In summary, our data demonstrate that STP dysregulation of Akt/GSK-3β pathway is associated with apoptosis, tau hyperphosphorylation, Aβ overproduction, synaptic dysfunction and cognitive decline. Restoration of Akt/GSK-3β pathway by GP might correct AD-like pathological changes and ameliorate cognitive deficits. Our study provides a promising application of GP for AD therapeutic strategy.

## MATERIALS AND METHODS

### Reagents

GP (Gynostemma Extract: Gypenoside IX, #CAS 80321-63-7, purity ≥99.92%) was provided by MedChemExpress (USA). STP is a cell inhibitor. It has been reported that STP can induce apoptosis at concentrations ranging from 0.2 to 1 μM [62, 63], which was provided by Abcam (ab146588, Cambridge, MA, USA). Table 1 lists the primary antibodies used in this study. IRDye 800CW-Conjugated secondary antibody (#ab216773, dilution 1:8000), used in Western blotting, was provided by Abcam.

**Table 1:** Primary antibodies.

**Antibodies**	**Specific**	**Type**	**Dilution**	**Source**
Bcl-2	Anti-Bcl-2	pAb	1:1000 for WB	ABclonal (A21592)
cleaved-caspase-3	Anti-cleaved-caspase-3	pAb	1:1000 for WB	Cell Signaling (#9661)
Bax	Anti-Bax	pAb	1:1000 for WB	Abcam (A12009)
p-AKT (473)	Anti-Phosphorylated AKT at Ser473	pAb	1:1000 for WB	ABclonal (AP1208)
p-AKT (308)	Anti-Phosphorylated tau at Tyr 308	mAb	1:1000 for WB	Santa cruz (sc-271964)
AKT	Anti-Total AKT	mAb	1:1000 for WB	Cell Signaling (#2920)
s9-GSK-3β	Anti-PhosphorylatedGSK-3βat Ser9	pAb	1:1000 for WB	ABclonal (AP0039)
GSK-3β	Anti-Glycogen synthase kinase-3β	pAb	1:1000 for WB	ABclonal (A11731)
pS199	Anti-Phosphorylated tau at Ser199	pAb	1:1000 for WB	ThermoFisher (44-734G)
pT231	Anti-Phosphorylated tau at Thr231	pAb	1:1000 for WB	ZENBIO (R381181)
pS262	Anti-Phosphorylated tau at Ser262	pAb	1:1000 for WB	Signalway Antibody (#21100)
pS396	Anti-Phosphorylated tau at Ser396	pAb	1:1000 for WB	Signalway Antibody (#21093)
pS404	Anti-Phosphorylated tau at Ser404	pAb	1:1000 for WB	Signalway Antibody (#21001)
AT8	Anti-Phosphorylated tau at Ser202/Thr205	mAb	1:1000 for WB	Thermo (MN1020)
tau5	Anti-Total tau	mAb	1:1000 for WB	Abcam (ab80579)
β-actin	Anti-β-actin	pAb	1:1000 for WB	ABclonal (AC026)
APP	Anti-C-terminal of APP	pAb	1:1000 for WB	Cell Signaling (E8B3O)
BACE1	Anti-BACE1	pAb	1:1000 for WB	Zen-BioScience (R381865)
MAP-2	Anti-MAP-2	pAb	1:200 for IF	Sigma-Aldrich (#M9942)
ADAM10	Anti-ADAM10	pAb	1:1000 for WB	Zen-BioScience (R381790)
PS1	Anti-PS1	pAb	1:1000 for WB	Zen-BioScience (R25440)
sAPPβ	Anti-sAPPβ	pAb	1:1000 for WB	Abcam (ab126732)
PSD95	Anti-PSD9	mAb	1:1000 for WB	Merck (ZMS1068)
synaptophysin	Synaptophysin (aa 250-350)	pAb	1:1000 for WB	Proteintech (17785-1-AP)
GluR2	Anti-Phosphorylated GluR2 at Ser 880	pAb	1:1000 for WB	Absin (abs123742)

Abbreviations: mAb: monoclonal antibody; pAb: polyclonal antibody; WB: Western blotting; IF: Immunofluoresence.

### Primary hippocampal neurons

Rat primary hippocampal neurons were obtained as described in our previous paper [64, 65].

For drug treatments, STP (dissolved in DMSO) was added to the culture medium and incubated with neurons for 24 h, and then the neurons were treated with GP (diluted according to the instruction) in different concentrations for 24 h.

### CCK8 assay

Cell Counting Kit 8 assay (CCK-8 assay) was employed to check the cell viability. About 5 × 10^3^ cells in a volume of 100 μL per well of 96-well plate were cultured in medium with 10% FBS, for four replicates. The cells were treated with different concentrations of STP without or with GP for different time. 10 μL CCK-8 reagent (ab228554, Abcam, Cambridge, MA, USA) was added into 90 μL DMEM to make the working solution. 100 μL working solution was added into per well and incubated for 2 h, then optical density detection.

### Animals and grouping

The study used male SD rats that were 2 months old and weighed around 250 ± 20 g. These rats were obtained from the Laboratory Animal Centre of Tongji Medical College, Huazhong University of Science and Technology. The rats were housed under standard laboratory conditions, including appropriate temperature and a 12/12-hour light/dark cycle. They had free access to water and food throughout the study. After a week of adapting to the environment, the rats were randomly divided into four groups including control group (CTR), STP injection group (STP group), GP treatment groups (STP+GP200, STP+GP400) (STP injection plus GP treatment at 200 or 400 mg/kg [66]). On the 1st day for surgery, rats were anesthetized with sodium pentobarbital at a dose of 0.15 ml per 100 g body weight. The sodium pentobarbital used was obtained from Sigma (Darmstadt, Germany). After anesthesia, the rats were placed on a stereotactic apparatus manufactured by Stoelting Corporation (Wood Dale, IL, USA). The STP group, and the STP+GP groups of the rats were injected with 3 μL STP (500 μM, dissolved in DMSO) in each side, 6 μL total in the bilateral hippocampi with a micro-syringe at a rate of 0.5 μL/min, at coordinates from bregma and dura of AP-4.0, L/R-2.0 and V-3.0 (in mm) [19]. The needle was left for 10 min after injection and then withdrawn slowly. Control rats were injected with the same volume DMSO. On the 3rd day, GP treatment groups were given 200 mg/kg and 400 mg/kg GP (dissolved in 0.9% saline) by gavage once a day for 30 days. The same volume of 0.9% sterile saline was used in the other groups. On the 33rd day, the rats were assessed by behavioral assessment of their learning and memory abilities. The brain samples were then collected for morphological testing, ELISA or Western blot analysis.

### Animal behavior tests

#### Open-field

Spontaneous exploratory motor activity and anxiety behaviors of rats were checked using open field (OF), as previously mentioned [67]. An open-ended black wooden box (with an internal field size of 100 × 100 cm × 70) is used. Each rat is tested once by placing it face to the wall and allowed to explore the environment for five minutes freely. At the end of each rat test, a wet cloth was used to wash and wipe the site and dry it with a dry cloth. We tested rat motor function by total movement distance.

#### Barnes maze task

The Barnes maze is a commonly used test to evaluate animal spatial learning and memory [68]. In this test, animals are trained to find a hidden escape chamber located beneath one of the holes on the perimeter of a disk. The disk is brightly illuminated to create an aversive stimulus. During the experiment, the animals are trained twice a day for four consecutive days. At the beginning of each trial, the animals are placed in the center of the maze, covered by a cylindrical starting chamber. After a 10-second delay, the starter chamber is lifted and the animal begins to search for the escape chamber. The experiment ends when the animal enters the escape chamber or after the predetermined 3-minute time limit. Before and after each experiment, all surfaces of the maze are cleaned to remove any potential olfactory cues left by the previous animals.

#### New object recognition

The novel object recognition test is used to assess learning and memory. The rats were put into a container with dimensions of 100 cm × 100 cm × 70 cm and allowed to freely explore the arena for five minutes to become acquainted with the environment. The next day, the rats are placed back into the arena from the same starting point. They are given five minutes to explore and familiarize themselves with two objects, object A and object B. After a 24-hour interval, object B (the familiar object) is replaced with a new object, object C. The rats are then given five minutes to explore both objects, object A and object C. The behavior of the rats during the exploration is recorded. The recognition index is calculated based on the time spent exploring the objects. The formula for calculating the recognition index is as follows: TA/(TA + TB): TB/(TA + TB): TC/(TA + TC).

### TUNEL assay

TUNEL staining was carried out to assess apoptosis using the Annexin V FITC/PI kit (Abbkine, China). Primary hippocampal neurons cultured on coverslips or brain slices were fixed in 4% paraformaldehyde for 30 minutes. The fixed cells or brain slices were then incubated in a solution of 0.5% Triton X-100 diluted in PBS for 10 minutes. The cells or brain slices in each group were incubated with the TUNEL solution at 4°C for 1 hour. After TUNEL staining, the cells or brain slices were incubated with DAPI for nuclear staining. The stained samples were visualized using a fluorescence microscope (Olympus, Tokyo, Japan). The resulting data were analyzed by counting the number of TUNEL-positive cells, which are indicative of apoptotic cells, as well as the total number of cells. The apoptosis rate was then calculated by dividing the number of TUNEL-positive cells by the total number of cells and multiplying the result by 100%.

### Immunofluorescence and dendrite morphological analysis

The cultured cells were prepared in the same way in TUNEL assay. The fixed cells were incubated in PBS containing 0.1% Triton X-100 and 5% BSA for 30 min at room temperature. The cells were then incubated with the primary antibody at 4°C overnight, followed by rinse for three times (5 min/time) in 0.01 M PBS. Next, the cells were incubated with the secondary antibodies from Jackson ImmunoResearch, a company based in West Grove, PA, USA, which were diluted at a 1:200 in PBS for 1.5 hours at room temperature, then the cells were rinsed three times in PBS. Finally, the coverslips were mounted onto the slides with DAPI to stain the nuclei of the cells. Laser confocal microscope (Zeiss 710, Germany) was used for image acquisition.

Neuronal dendrite morphology was analyzed with ImageJ software. The saved grayscale image was opened in ImageJ, and the ruler was set. The “Neuron J” patch was used to trace the dendrites and outline the neuronal structure, and then, the picture was saved. Lastly, the saved picture was opened again, draw the distance from the cell body to the farthest dendrite, and click “Sholl Analysis” to get the relevant measurements of the neuron dendrites. The data were saved for subsequent analysis.

### Western blotting

Protein samples of primary hippocampal neurons and tissues were prepared according to the method in our previous paper [65]. 10 μg protein from hippocampal tissue and primary hippocampal neurons were separated using SDS-PAGE and transferred onto a nitrocellulose membrane (Amersham Biosciences, Piscataway, NJ, USA). Next, the membranes were subjected to overnight incubation at 4°C with primary antibodies. After rinsing with TBST for three times, the membranes were incubated with secondary antibodies. The results were shown using the detection reagent and analyzed using ImageJ software (Rawak Software, Stuttgart, Germany).

### ELISA for Aβ1–42 and Aβ40 levels

Aβ40 and Aβ42 levels are commonly performed using a sandwich ELISA kit. Primary hippocampal neurons or hippocampal tissues were homogenized using PBS with the addition of a protease inhibitor PMSF at a concentration of 100 mg/ml. After homogenization by repeated freezing and thawing to break down the cells and tissues, the samples were centrifuged at 2.5 × 10^3^ g rpm for 20 min. The supernatant was then incorporated into a 96-well plate. The ELISA assay followed the protocol provided by the manufacturer, Elab-science Biotechnology, located in Wuhan, China.

### Nissl staining

After perfusion and fixation, the brains were sectioned into slices with a thickness of 30 μm. These sections were then mounted on slides that had been coated with gelatin. To visualize the neurons, Nissl staining solution was used. The staining procedure followed the instructions provided by the manufacturer, BeoTeam Biotechnology Co., Ltd., located in Shanghai, China.

### Golgi staining

Golgi staining is a technique employed to visualize dendritic spines. The FD Rapid Golgi Staining Kit PK401, developed by FD Neuro Technologies, Inc. in Columbia, MO, USA, is one of the commonly used kits for this purpose. Brain sections were stained following the manufacturer’s instructions provided. The hippocampus slices were imaged by 10×, 20× and 40× objectives using Olympus VS120.

### Statistical analyses

Statistical analyses in the study were carried out by GraphPad Prism 9 statistical software. The data were presented as mean ± SEM (standard error of the mean). One-way analysis of variance (ANOVA) was used to assess the differences among groups, *P*-value was equal to or less than 0.05, the observed differences were considered statistically significant.

### Data availability statement

All data used in this study are available from the corresponding authors on reasonable request.
